# Network hypermotifs in biological systems: structure, dynamics, and functional implications

**DOI:** 10.1038/s41540-026-00701-7

**Published:** 2026-04-15

**Authors:** Moirangthem Sailash Singh, Priyan Bhattacharya, Karthik Raman

**Affiliations:** 1 Department of Data Science and AI, Wadhwani School of Data Science and AI, IIT Madras, Chennai, 600 036 India; 2 Centre for Integrative Biology and Systems Medicine (IBSE), Wadhwani School of Data Science and AI, IIT Madras, Chennai, 600 036 India; 3 AstraZeneca India Private Limited, Bengaluru, India

**Keywords:** Computational biology and bioinformatics, Systems biology

## Abstract

Biological networks exhibit complex structural organization, from motifs, which are recurring patterns of gene, protein, and molecular interactions, to higher order assemblies such as hypermotifs. Hypermotifs are statistically significant higher order assemblies formed through interactions and combinations of motifs, giving rise to collective structural and functional properties. This review discusses hypermotifs in biological networks, focusing on their structure, dynamics, functions, and patterns such as motif clustering and motif generalizations.

## Introduction

Biological networks exhibit rich structural patterns that often reflect underlying cellular functions. Over the past two decades, analysing these networks through network motifs—small, statistically enriched wiring patterns that recur more often than expected in appropriate null models—has advanced our understanding of signal processing, decision-making, and robustness in complex biological systems^1,2^. However, there exists a wide range of functionalities that can only be described through specific interconnections of the motifs. This motivates the concept of network *hypermotifs*, which are composite structures formed by interconnected motifs that give rise to novel dynamical behaviours not explainable by isolated motifs^3,4^.

In this review, we use “hypermotifs” to denote statistically significant mesoscale assemblies in which multiple motifs interconnect in specific ways to generate collective structural and functional properties beyond those of individual motifs^3,4^. Crucially, this perspective highlights which motif interconnections are sufficient to generate emergent dynamics. This distinction is evident in practice. For example, in neuronal circuits, recurring combinations of mutual-feedback motifs can support the transmission of oscillations and phase synchronization across coupled subcircuits, producing coordinated activity that isolated motifs cannot achieve^4^. Hypermotifs are also central to biological decision-making that is robust and history-dependent^3^. Hypermotif formed by couplings of feedback and feed-forward motifs have been linked to robust, history-dependent cellular decision-making, as specific interconnection patterns can modulate multistability, alter switching thresholds, and reshape the state-transition landscape of gene regulatory programs^2,5,6^. Together, these examples show that hypermotifs provide an intermediate scale between single motifs and full networks, where wiring structure can be linked directly to qualitative dynamics^3,4^.

This review discusses the current understanding of network hypermotifs in biological systems, with a focus on three key network types: regulatory networks, which govern gene expression; signalling networks, which transmit cellular signals; and developmental networks, which guide differentiation events. In these biological networks, we examine the structural formation, dynamic properties, and functional roles of network hypermotifs. We outline recent advances in modelling frameworks, AI/ML-driven tools, and graph-theoretical methods that facilitate their discovery and analysis. Furthermore, we highlight emerging approaches in data-driven motif identification, ensemble simulation techniques, and network embedding, which are reshaping our understanding of structure–function relationships in complex biological systems. To study hypermotifs systematically, we briefly revisit the canonical *network motifs* that serve as the elementary building blocks of hypermotifs. Although hypermotifs emphasize mesoscale organization and emergent dynamics arising from motif interconnections, these effects are best interpreted relative to the well-established dynamical roles of the constituent motifs. Accordingly, the next section provides a concise motif-level baseline and shared terminology, which we then use to describe how specific motif combinations and interactions generate collective behaviours in biological networks.

## Network Motifs

Network motifs are recurrent, functionally significant patterns in biological networks. Each motif performs distinct information-processing roles, which have been extensively analysed through mathematical modelling and validated by dynamic experiments in living cells^2^. We begin by briefly reviewing the most common motifs observed in biological systems. The simplest motif is the single-input regulation, in which a transcription factor ${\mathcal{X}}$ directly controls the expression of a target gene ${\mathcal{Y}}$ without any additional regulatory interactions. Upon activation of transcription, the concentration of ${\mathcal{Y}}$ rises and eventually stabilises at a steady-state level. Conversely, when production stops, the concentration of ${\mathcal{Y}}$ decays exponentially. The rates at which ${\mathcal{Y}}$ increases or decreases depend on its underlying production and degradation constants^7^. Negative autoregulation (NAR) is another fundamental regulatory motif in which a transcription factor inhibits the expression of its own gene^2,8,9^. NAR motifs are very common in the repressors in *E. coli*^2,10^ and in many eukaryotic repressors^11^. NAR enables a gene to reach its steady-state expression level faster than in the absence of feedback^7^. Moreover, NAR reduces cell-to-cell variability (extrinsic and intrinsic noise) in protein levels. In positive autoregulation (PAR) motifs, the transcription factor promotes its own expression, which tends to slow down the response time^12,13^ and increase cell-to-cell variability in gene expression^14,15^. Among the most studied motifs are: (i) feed-forward loops (FFLs), which can act as pulse generators, response accelerators, sign-sensitive filters, and appear in hundreds of gene systems in *E. coli*^16,17^ and yeast^1,11^, as well as in other organisms^18–23^; (ii) feedback loops, where negative feedback contributes to homeostasis^24–26^ and oscillations^27,28^, while positive feedback enables bistability and memory^29^; and (iii) mutual repression (toggle switches), which underlie binary decision-making in differentiation. Table 1 summarises key network motifs commonly found in biological systems, along with their characteristic topologies and associated functional roles. These motifs provide a foundation for understanding regulatory logic in transcriptional and signalling networks.

**Table 1:** Gene regulatory motifs and their biological roles

Motif	Topology	Biological Role
Simple Regulation		Transcription factor ${\mathcal{X}}$ regulates gene ${\mathcal{X}}$ with no additional interactions
Negative autoregulation (NAR)		Transcription factor ${\mathcal{X}}$ represses the transcription of its own gene. NAR speeds up the response time of gene circuits and can reduce cell-cell variation in protein levels
Positive autoregulation (PAR)		Transcription factor enhances its own rate of production. Response times are slowed and variation is usually enhanced.
Coherent Feedforward Loops		Coherent type 1 FFL (C1-FFL) occurs much more frequently than the other three types. This type FFL can act as sign-sensitive delay element and a persistence detector.
Incoherent Feedforward Loops		The Incoherent type 1 FFL (I1-FFL) is a pulse generator and response accelerator and occurs more frequently than the other types
Feedback Loops		Double-positive loop: Two activators activate each other and has two steady states: either both ${\mathcal{X}}$ and ${\mathcal{Y}}$ are OFF, or both are ON. Double-negative loop: Two repressors repress each other and has different steady states: either ${\mathcal{X}}$ is On and ${\mathcal{Y}}$ is OFF, or the opposite.

## Hypermotifs in Biological Networks

**Fig. 1:**
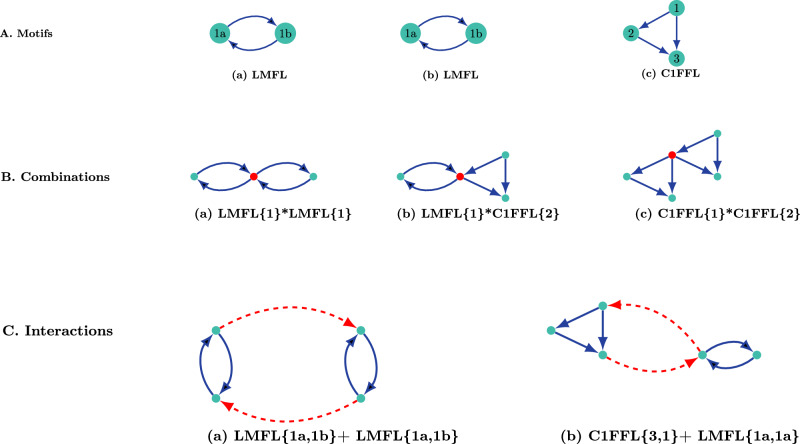
Schematic representation of motif combinations and interactions with shared-node and edge-linking notation. **A** The Lock-ON mutual feedback loop (LMFL) in figures (a) and (b) and the coherent type 1 FFL (C1FFL) in fig. (c). **B** Combination of motifs, *A*{*i*}*B{*j*}, where the two motifs share at least one node and {*i*}, {*j*} is the set of nodes each motif is sharing. The shared nodes are marked in red. **C** Interaction of motifs *A* and *B* where the two motifs are linked through at least one edge, *A*{*i*, *j*} + *B*{*k*, *l*}, where *i* and *k* are the set of sender nodes, and *j* and *l* are the set of receiver nodes from motifs *A* and *B*, respectively. The linking edges are marked in red.

Hypermotifs describe statistically significant mesoscale patterns formed by the combination or interaction of multiple network motifs, giving rise to collective structural and functional properties that cannot be inferred from the constituent motifs in isolation^3^. A combination of two motifs *A* and *B* occurs when the two motifs overlap by sharing at least one node. Such combinations are denoted by *Af*_i_*Bf_*j*_, where *f*_*i*_ and *f*_*j*_ denote the sets of nodes shared by motifs *A* and *B*, respectively. An interaction between motifs *A* and *B* occurs when the two motifs are connected by at least one directed edge whose endpoints belong to different motifs. This is denoted by *Af*_*i*,*j*_ + *Bf*_*k*,*l*_, where *i* and *k* denote the sets of sender (source) nodes in motifs *A* and *B*, respectively, and *j* and *l* denote the corresponding sets of receiver (target) nodes^3^. Figure 1 shows examples of hypermotifs formed due to the combination and interaction of motifs. We now formalize these notions in graph-theoretic terms by defining motifs as induced subgraphs of a directed network and characterizing hypermotifs as induced subgraphs formed by unions of motifs coupled through overlap or inter-motif edges.

Let *G* = (*V*, *E*) be a (directed) network, and let ${\mathcal{M}}=\{{M}_{1},{M}_{2},\ldots ,{M}_{N}\}$ denote the set of network motifs identified in *G*, where each motif *M*_*i*_ is defined as an induced subgraph of *G* belonging to a given motif isomorphism class. A *hypermotif* is an induced subgraph *H* ⊆ *G* composed of two or more motifs from ${\mathcal{M}}$ that are interconnected through either *combination* or *interaction*. In a combination, two motifs *M*_*i*_ and *M*_*j*_ overlap by sharing at least one vertex, i.e., $V({M}_{i})\cap V({M}_{j})\ne {\rm{\varnothing }}$, whereas two motifs *M*_*i*_ and *M*_*j*_ form an interaction if they are vertex-disjoint, $V({M}_{i})\cap V({M}_{j})={\rm{\varnothing }}$, and there exists at least one edge (*u*, *v*) ∈ *E*7D1with*u* ∈ *V*(*M*_*i*_), *v* ∈ *V*(*M*_*j*_), or vice versa. The hypermotif *H* is defined as the induced subgraph on the union of the vertices of all participating motifs, $H=G\,\left[{\bigcup }_{k}V({M}_{k})\right]$. With this formal definition in place, we now focus on the study of hypermotifs in three major biological settings—transcriptional, neuronal, and signalling networks as follows.

### Transcriptional network

**Fig. 2:**
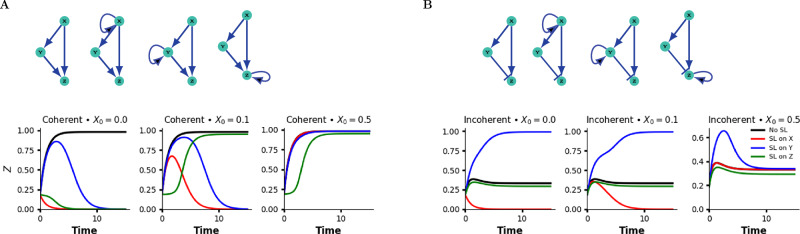
Emergent dynamical behaviour arising from different motif combinations. **A** Combinations of self-loop and coherent FFLs. **B** Combinations of self-loop and incoherent FFLs leading to distinct output dynamical behaviour of node ${\mathcal{Z}}$.

In the E. coli transcriptional network where nodes represent transcription factors and their target genes, and edges denote regulatory interactions, self-loop and FFL motifs are often combined such that the intermediate node of the FFL exhibits autoregulation^16^. When examining combinations of self-loops with FFL circuits, an important question is whether the functional outcome depends on which node in the FFL carries the self-regulation. To address this, Adler et al.^3^ compared the dynamics of a canonical FFL (without self-loops) to the cases in which the input, intermediate, or output node was autoregulated. Adding self-loops generally introduced bistability into the FFL’s response, such that the final output depended on the initial level of the input. Interestingly, FFLs in which the output node was autoregulated were less sensitive to the input’s initial state (Fig. 2 Left Panel). In incoherent FFLs, self-loops on the intermediate repressor (${\mathcal{Y}}$) produced striking non-trivial behaviours. With high initial input, the output displayed the familiar pulsatile response of a standard incoherent FFL. However, with low initial input, the output ${\mathcal{Z}}$ rises to a delayed but sustained high steady-state. Notably, this emergent high-output state was possible only when the intermediate node is autoregulated (Fig. 2 Right Panel). This combination of a self-loop with an FFL may explain why, in the E. coli network, the intermediate node often has a self-loop, as it creates bistable responses that adjust how target genes respond to input signals.

### Neuronal network

**Fig. 3:**
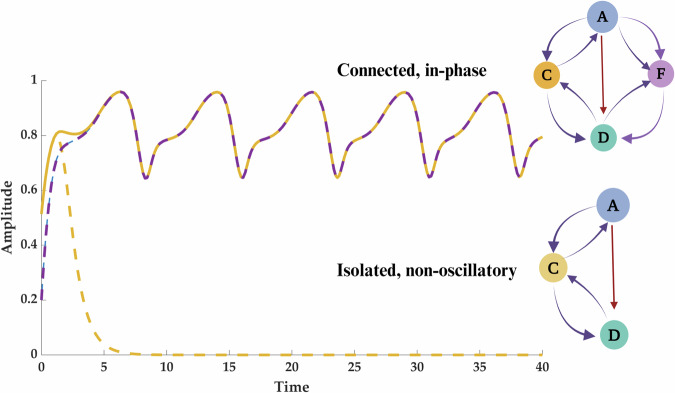
Dynamical behaviour of overrepresented double mutual feedback circuits in *C. elegans.* In this circuit, ${\mathcal{A}}$ inhibits ${\mathcal{D}}$. In the top figure, ${\mathcal{F}}$ and ${\mathcal{C}}$ exhibit in phase synchronization. In the corresponding isolated ${\mathcal{A}}$, ${\mathcal{C}}$, and ${\mathcal{D}}$ circuit with the same parameters, ${\mathcal{C}}$ decays to zero without oscillations.

The neuronal network of *C. elegans*, where the edges are the synaptic strengths between the neurons, contains six distinct motifs, including the FFL and five different forms of mutual feedback circuits^1,30^. Similar overrepresented combinations of motifs are also found in other neuronal systems^31,32^. In these networks, the over-represented combinations show that the hypermotifs are formed in a layered structure, where the output of one motif serves as the input to another motif. The mutual feedback circuits, however, are not connected in a layered manner. A hypermotif consisting of two pairs of mutually interacting neurons (${\mathcal{X}}$ and ${\mathcal{Y}}$, ${\mathcal{Y}}$ and ${\mathcal{Z}}$), with a one-way interaction from ${\mathcal{X}}$ to ${\mathcal{Z}}$, frequently appears in neuronal networks, with the one-direction edge from ${\mathcal{X}}$ to ${\mathcal{Z}}$ shared between the two motifs. The interactions among the motifs are excitatory and therefore, each motif functions as a generalised Lock ON circuit, supporting bistability, where ${\mathcal{X}}$, ${\mathcal{Y}}$, and ${\mathcal{Z}}$ are either all ON or all OFF. Coupling two such motifs links the behaviour of shared nodes such as ${\mathcal{Y}}$ and ${\mathcal{W}}$, enabling coordinated bistable switching or sequential pulsatile dynamics. When ${\mathcal{X}}$ inhibits ${\mathcal{Z}}$, each double mutual feedback motif can produce oscillations or settle into an OFF state depending on parameter values. When two such inhibitory motifs are connected through the shared ${\mathcal{X}}$ to ${\mathcal{Z}}$ edge, oscillations can propagate from one motif to the other, inducing synchronised activity and phase alignment even in circuits that would not oscillate independently, as shown in Fig. 3.

### Hypermotifs in signalling networks

Hypermotifs help explain how signaling networks achieve timing, robustness, and decision-making beyond what isolated motifs can provide. In temporally staged programs such as *Bacillus subtilis* sporulation, cascaded feed-forward control supports ordered waves of gene expression, motivating the view that chained FFL-like modules can act as higher-order timing devices^6^. In mammalian pathways, motif-composition analyses of TGF-*β*-driven EMT identify a layered, coherent type-1 feed-forward logic that preferentially transmits sustained stimulation while filtering transient fluctuations, thereby stabilizing the activation of EMT regulators such as *SNAIL* and *ZEB*^5^. More generally, coupling feedback motifs into hypermotif-like composites can yield robust bistable switches that encode cellular memory and enable fate commitment after an initiating cue decays^33^. In neuronal signaling, higher-order integration of convergent inputs can implement coincidence detection, as illustrated by microcircuit mechanisms in the medial superior olive^34^. A complementary, higher-order perspective on signaling architecture is provided by *signaling hypergraphs*, which represent biochemical reactions and pathway logic as hyperedges connecting sets of reactants to sets of products, thereby capturing many-to-many interactions that are obscured in pairwise graphs. Ritz et al. formalize pathway inference directly in this representation by asking which proteins and interactions must participate to elicit a specified downstream response, and relate this query to computing a *shortest acyclic B-hyperpath* in a signaling hypergraph. This illustrates how hypergraph structure can encode mechanistic constraints more faithfully than conventional graphs^35^. In the context of hypermotifs, hypergraphs provide a natural way to represent signaling networks and to define and detect recurring higher-order building blocks. Thus, signaling hypergraphs do not replace hypermotifs; rather, they provide an *expressive representation* in which hypermotifs correspond to reusable higher-order pathway fragments, and in which algorithmic primitives such as shortest B-hyperpaths can be leveraged to identify minimal mechanistic substructures that instantiate particular hypermotif functions^35^. Hypergraph-based analyses further link resilience of flow-weighted biological networks to a small set of critical hypermotifs, whose disruption can precipitate system-wide failures^36^.

**Fig. 4:**
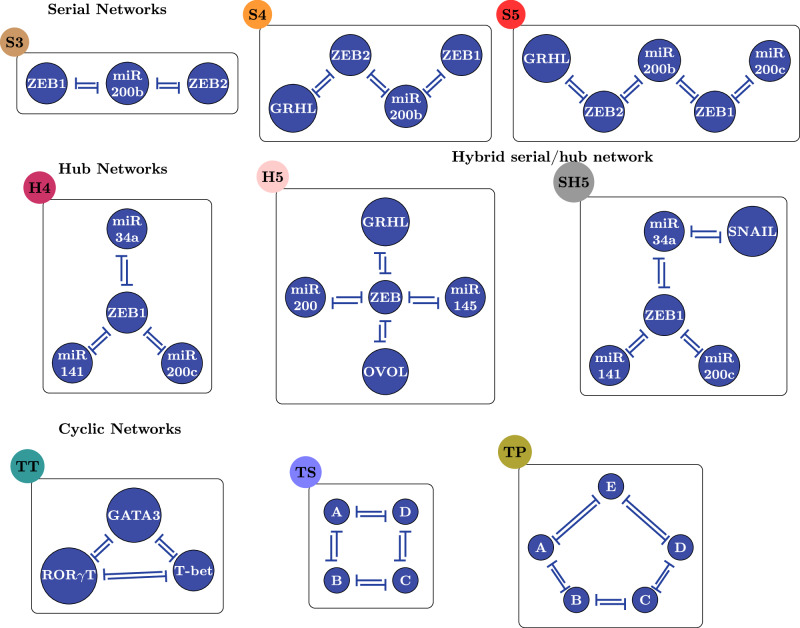
Illustration of different classes of high-dimensional feedback architectures. Adapted from Sai Bhavani and Palanisamy (2023)^5^, licensed under CC BY 4.0. Serial networks consist of toggle switches linked sequentially, while hub networks feature multiple nodes converging on a single central node. Cyclic networks form closed feedback loops among nodes. Each topology is colour-coded and labelled to indicate its structure and size (e.g., S3 for a 3-node Serial network, H4 for a 4-node Hub network). A hybrid Serial-Hub network with five nodes is denoted SH5. Cyclic variants include the toggle triad (TT, 3 nodes), toggle square (TS, 4 nodes), and toggle polygon (TP, 5 nodes). Networks with annotated nodes correspond to biological systems, whereas unannotated ones represent synthetic designs. Edges ending with bars denote inhibitory interactions, and reciprocal inhibition between two nodes defines a toggle switch.

**Fig. 5:**
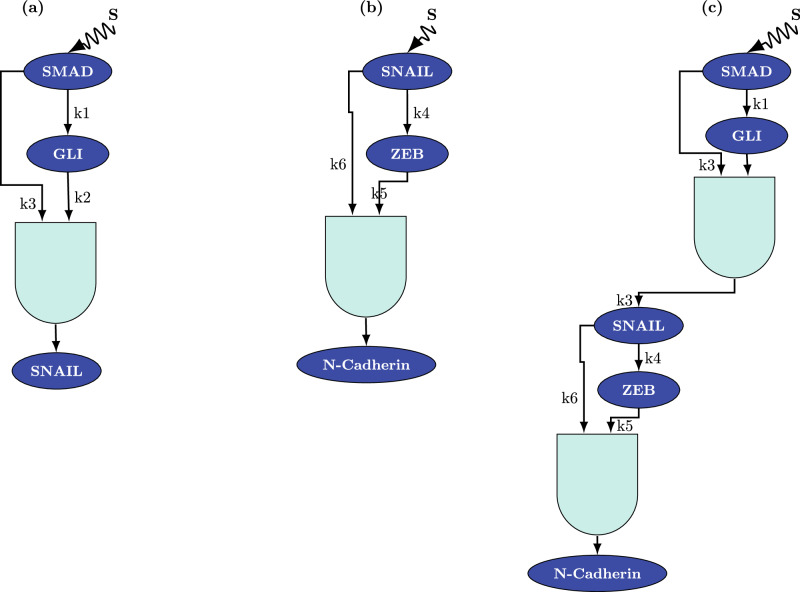
Network motifs from the assembled network that critically regulate the process of EMT induced by TGF-*β.* Adapted from Sai Bhavani and Palanisamy (2023)^5^, licensed under CC BY 4.0. **a** C1FFL regulation of SNAIL. **b** C1FFL regulation of N-cadherin. **c** Combination of C1FFLs in regulating the expression of N-cadherin.

One biologically prevalent class of hypermotifs in signalling networks is formed by coupled feedback architectures. For example, compared to a single positive feedback loop (PFL), two interconnected PFLs significantly expand the range of cellular conditions that support bistability^37,38^. Interconnections of several positive feedback loops are common in biological systems. Examples include stepwise lineage decisions in *CD*4^+^ T cells, cell cycle regulation, neuronal fate determination, calcium signalling, B cell fate specification, and epithelial-mesenchymal transition (EMT)^37–46^. A recent study^47^ identified topologically distinct hypermotifs composed of interconnected PFLs embedded within complex biological networks, particularly those involved in EMT-driven carcinoma phenotypic transitions and *CD*4^+^ T cell differentiation^48–53^. The hypermotifs fall into three global topological categories (Fig. 4): (1) serial (S3-S5), where motifs are linked sequentially; (2) hub-like (H4-H5), where multiple motifs converge on a central node; and (3) cyclic (TT, TS, TP), where motifs form closed loops. A fundamental unit in these architectures is the toggle switch, a PFL formed by two mutually antagonistic genes. These hypermotifs control key biological processes by driving cell fate transitions. For instance, in the S3 serial network, mutual repression between miR-200 and ZEB drives carcinoma cells into three phenotypes: epithelial (high miR-200, low ZEB), mesenchymal (low miR-200, high ZEB), and hybrid epithelial-mesenchymal (medium miR-200, medium ZEB)^49^. Similarly, the TT cyclic network of ROR*γ*T, GATA3, and T-bet regulates naive *CD*4^+^ T cells into Th1 (high T-bet), Th2 (high GATA3), or Th17 (high ROR*γ*T) states^54^. Connecting multiple toggle switches in series, as in S5, gives rise to a wide range of alternative stable states, highlighting the functional importance of hypermotif topology. In the context of EMT, the TGF-*β* signalling network illustrates a layered hypermotif architecture controlling N-cadherin expression^3,5^. TGF-*β* activates both SMAD-dependent and SMAD-independent pathways, converging on the transcription factor SNAIL. Two coherent type-1 FFLs (C1FFLs) are sequentially arranged (Fig. 5): the first regulates SNAIL directly and indirectly via GLI (Fig. 5a), and the second regulates N-cadherin directly and indirectly via ZEB (Fig. 5b). Combined, these C1FFLs form a cascade-like hypermotif, where SNAIL acts as an intermediate integrator linking upstream signals to downstream EMT markers (Fig. 5c).

The core EMT network involves two mutually inhibitory loops: miR-34/SNAIL and miR-200/ZEB. Tian et al.^55^ suggest both loops act as bistable switches driving EMT transitions. While Jolly et al.^56^ argue that miR-34/SNAIL serves as a monostable noise filter, and only miR-200/ZEB, influenced by SNAIL, functions as a tristable switch enabling epithelial, mesenchymal, and hybrid states. The two models of the EMT network differ in their explanation of partial EMT due to distinct assumptions^56^.

### Recurrent hyper-motif circuits in developmental programs

**Fig. 6:**
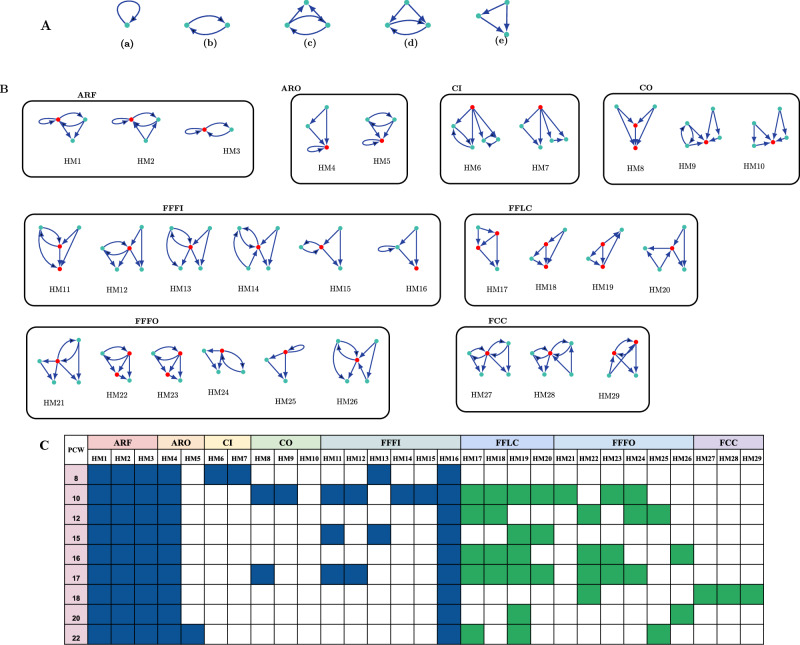
Development related three node motifs and their interconnected hypermotif topologies exhibit stage specific enrichment patterns across PCWs. Concept adapted from Adler and Medzhitov (2022)^3^, licensed under CC BY 4.0. **A** Common three-node circuit motifs found in development-related genes. **B** Enriched hypermotif topologies formed by interconnecting motifs. **C** Over- and under-represented hypermotifs across developmental stages (PCWs). Columns indicate different hypermotif topologies, and rows represent successive time points. Blue squares denote positive enrichment, while green squares indicate negative enrichment.

Motifs and hypermotifs play a crucial role in the developmental stages of various biological systems, forming the underlying design principles that enable cells to develop robust and diverse spatial and temporal patterns. The cells rely on secreted and sensed communication signals to regulate their fate decisions^57^. These signals, known as morphogens, form gradients and spatial patterns that provide positional cues, enabling cells to respond appropriately^58–62^. Several morphogen families are central to embryonic patterning, including bone morphogenetic proteins (BMPs)^63,64^, fibroblast growth factors (FGFs)^65^, Hedgehog (HH)^66^, WNT^67,68^, and retinoic acid^69^. Their production and sensing are controlled by key transcription factor (TF) families such as HOX, GATA, ETS, SOX, PAX, TBOX, FOX, and E-proteins^70–78^. Together, morphogens and TFs regulate each other through intracellular feedback circuits and intercellular communication networks, creating a highly interconnected gene regulatory system. The studies in ref. ^4^ utilise single-cell gene expression data to infer the motifs and hypermotifs that regulate developmental programs across various stages of human intestinal development. Specifically, for any pair of network motifs, the study examined two modes of interconnection to form a hypermotif: sharing at least one common node between the motifs, and linking them through at least one edge within the network. The network comprises development-related genes, including developmental transcription factors, morphogen ligands, their antagonists, receptors, and co-receptors, identified from single-cell RNA sequencing (scRNAseq) data of the human intestine across nine time points, spanning 8 to 22 post-conceptual weeks (PCWs)^79^. In this network, the five most common three-node circuit motifs, which are also found in the developmental programs of other organisms, are (a) autoregulation, (b) mutual feedback loop, (c) regulated feedback loop, (d) regulating feedback loop, and (e) FFL, as shown in Fig. 6A.

The hypermotifs formed by interconnecting these motifs are classified into several categories as: (a) Autoregulation and Feedback (ARF), (b) Autoregulation and Outputs (ARO), (c) Common Inputs (CI), (d) Common Outputs (CO), (e) Feedback and FFL through the FFL’s intermediate node (FFFI), (f) FFL Cascades (FFLC), (g) Feedback and FFL through the FFL’s input or output node (FFFO), and (h) Feedback Chains of Cascades (FCC). While some of these hypermotifs appear across all developmental stages, others are present only at specific time points. The table in Fig. 6C illustrates the distribution of hypermotifs during development (PCWs), where rows correspond to different hypermotif topologies and columns represent successive time points. Blue squares highlight positively enriched hypermotifs, while green squares mark those that are under-represented. The most enriched hypermotif topologies are observed at 10 PCW, which corresponds to the formation of the crypt to villus axis (Fig. 6B). Autoregulation and mutual feedback loops do not combine with the input nodes of feed-forward loops (FFLs). Additionally, combinations involving multiple feedback circuits are absent at 18 PCW, and cascades of FFLs are excluded at several time points.

## Functional Implications

The analysis of motifs and hypermotifs in biological and other complex networks is made feasible by the principle of modularity, which is defined as the ability to decompose a system into semi-independent functional units. This structural characteristic enables parts of the network to operate with a degree of independence, particularly in the context of approximate dynamics. Dynamical features such as robustness and multistability are known to favour the spontaneous emergence of modular structures, especially under variable environmental conditions. As a result, the interactions among individual motifs give rise to a variety of emergent dynamic behaviours. In this section, we explore several representative hypermotifs and the associated dynamical properties observed in both natural and engineered biological systems.

### Bistability and Multistability

**Fig. 7:**
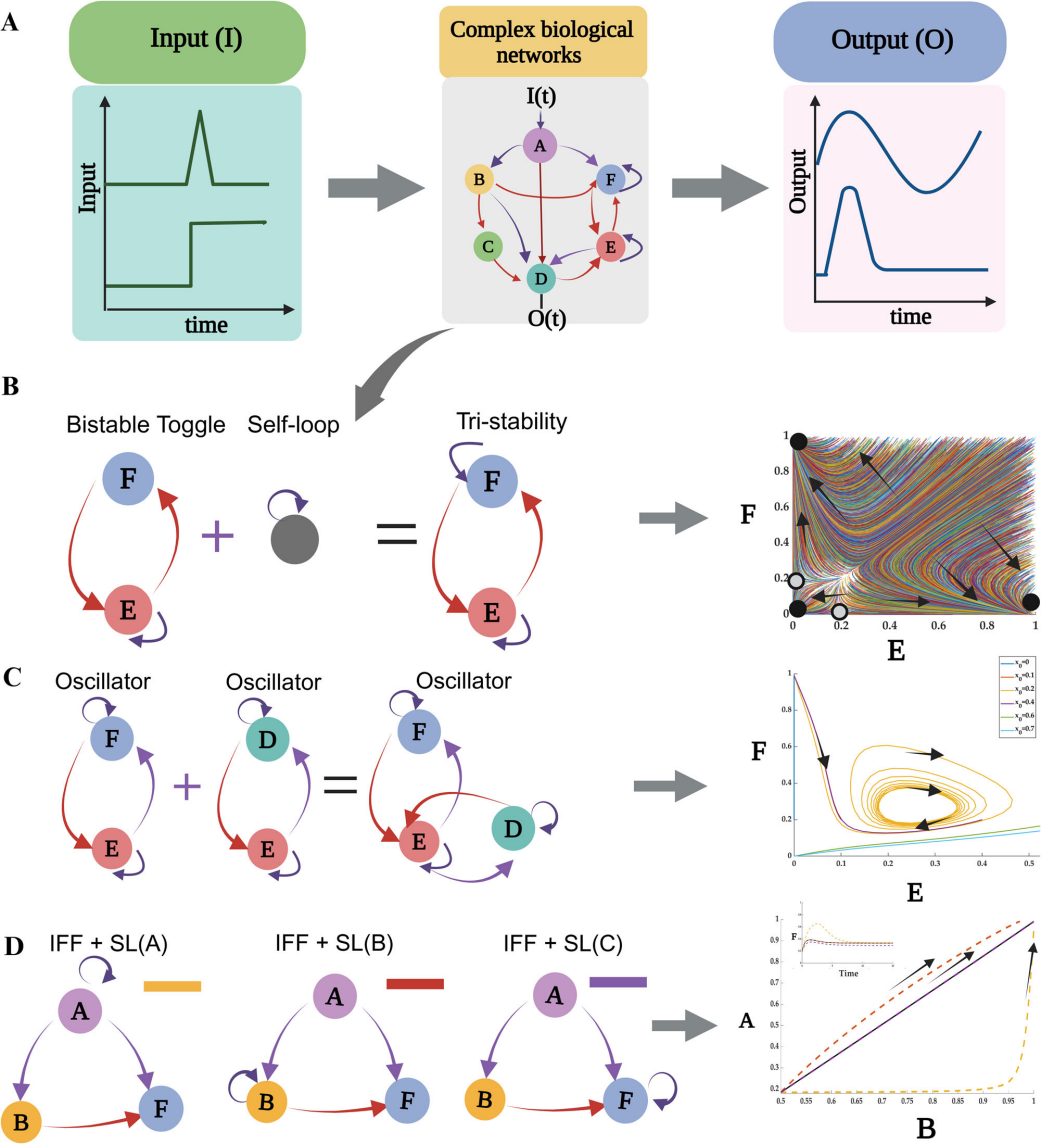
Demystifying the complex networks with hypermotifs. **A** Understanding the response of a complex biological network to a specific input. The red and blue arrows represent inhibition and activation, respectively. **B** The possible hypermotif constructed by combining bistable switches with a self-loop to provide tristability. **C** Depicts the possibility of providing sustained, robust oscillation by combining two elementary oscillatory motifs. **D** On the other hand, the possibility of emergent response for the combination of incoherent feed-forward (IFF) and self-loop depends on the specific node on which the self-loop is mounted. In isolated condition, the 2 dimensional (considering nodes ${\mathcal{A}}$ and ${\mathcal{B}}$) phase renders a straight. Although this feature remains conserved with self-loops at nodes ${\mathcal{A}}$ and ${\mathcal{C}}$, a highly nonlinear relationship emerges in the phase space when the self-loop is mounted on node ${\mathcal{B}}$.

The integration of positive self-loops into other circuit motifs promotes bistability in the self-activating node. For instance, adding self-loops to a toggle switch results in a new stable OFF state where both nodes remain inactive (Fig. 7 (Second Row)). Similarly, oscillator circuits with self-regulation in both nodes exhibit two additional steady states alongside oscillatory behaviour: a complete OFF state and a state in which only the activator (Y) is active, since the repressor (X) cannot sustain activation independently^3^. Conversely, negative self-loops do not promote bistability but can accelerate the circuit’s dynamic response^7^. Interestingly, in incoherent FFLs, a positive self-loop on the intermediate repressor (Y) results in non-trivial dynamics: high initial input produces a pulse-like output similar to a standard incoherent FFL, while low initial input leads to a delayed steady state output^3^. This occurs because the self-regulation causes ${\mathcal{Y}}$ to decline, thus lifting the repression on the output node ${\mathcal{Z}}$ and allowing its activation. Crucially, this behaviour arises only when the intermediate node is positively autoregulated. Thus, self-loops can introduce threshold-like behaviour in coupled dynamic circuits. This mechanism may explain why FFLs in the E. coli transcriptional network are often combined with self-loops at the intermediate node, potentially enabling E. coli to implement bistable gene expression programs that respond sensitively to the strength of incoming signals.

### Sensitivity to initial conditions

**Fig. 8:**
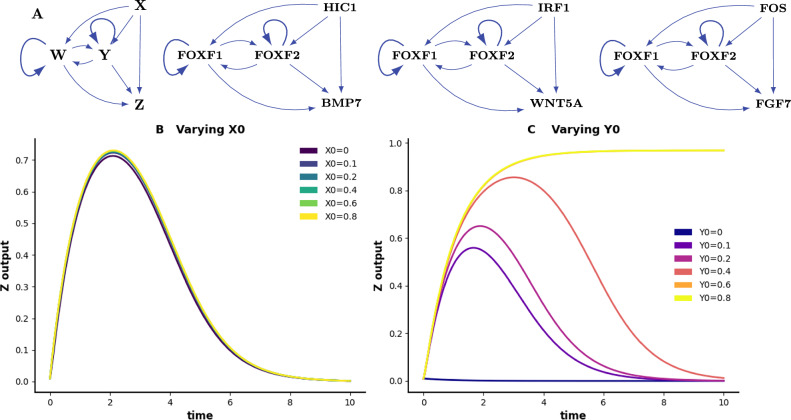
An enriched developmental hypermotif exhibits distinct output dynamics of ${\mathcal{Z}}$ under different initial conditions. Adapted from Adler and Medzhitov (2022)^3^, licensed under CC BY 4.0. **A** An enriched hyper-motif circuit in which the input signal ${\mathcal{X}}$ activates the output gene ${\mathcal{Z}}$ as well as the transcription factors ${\mathcal{Y}}$ and ${\mathcal{W}}$, which in turn also stimulate ${\mathcal{Z}}$ and examples of this hyper-motif within developmental networks. **B** Dynamic response of the output gene ${\mathcal{Z}}$, with the plotted trajectories corresponding to varying initial conditions of the input signal ${\mathcal{X}}$ or transcription factor ${\mathcal{Y}}$ in (**C**).

For distinct combinations of oscillatory circuits, variations in initial conditions lead each configuration to exhibit distinct dynamic behaviours and converge to different steady states (see Fig. 7, Third Row). The hypermotif formed by connecting feedback circuits to FFL through the FFL’s intermediate nodes is shown in Fig. 8A and in Fig. 6B (HM11 to HM16). This hypermotif is commonly found in the regulatory networks of stromal and epithelial cells during development, where it directly regulates the expression of morphogen ligands, receptors, and key transcription factors involved in developmental processes^4^. An interesting property of this hypermotif is that the dynamical response of the output ${\mathcal{Z}}$ depends on which input node is activated. In Fig. 8A, the output gene is ${\mathcal{Z}}$ and there are two types of input signals affecting ${\mathcal{Z}}$. The first type is the FFL’s input ${\mathcal{X}}$, and second type are the ${\mathcal{Y}}$/${\mathcal{W}}$ TFs, which mutually regulate each other. While the feed-forward input ${\mathcal{X}}$ is necessary to activate the output, its initial or final levels do not affect the dynamics of ${\mathcal{Z}}$ (Fig. 8B). In contrast, the initial levels of ${\mathcal{Y}}$ and ${\mathcal{W}}$ strongly influence the temporal behaviour of the output, allowing the circuit to generate distinct responses to varying levels of input using a simple regulatory circuit (Fig. 8C). This sensitivity to ${\mathcal{Y}}$ or W allows a simple regulatory circuit to produce distinct responses to varying input levels, supporting the interpretation of morphogen gradients, for example.

### Pulsating Response

Coupling oscillator circuits with FFLs results in distinct dynamical responses. Specifically, an oscillator circuit with a coherent FFL yields the features of a pulsating response, while coupling with an incoherent FFL results in low and high initial levels^3^ (see Fig. 7, Fourth Row).

### Leveraging structural control for efficacious therapy design: Feedback vertex sets

The aforementioned sections establish an instrumental role of network structure in determining the response of the network output in the presence (or absense) of the external stimuli. This inspires an interesting problem of leveraging the structure of a network for a reliable prediction of its response. From an engineering perspective, the problem of determining a structure-function relationship can have a potential of translational impact in the context of serious diseases such as solid tumors. For instance, the application to chemotherapy is often met with resistance for patients with advanced-stage solid tumors. This can be explained by looking at the tumor cell signalling network that governs the proliferation, and growth of the tumor cells. Interestingly, the phenomenon of chemo-resistance can be cast as the well-known ‘adaptation’ behavior against the external stimuli (the chemo drug in this case)^80^. Further, the previous studies have proved that the existence of negative feedback with buffer nodes or feedforward incoherency is a necessary condition for any biological network to provide perfect adaptation^26,81^. A systematic analysis of the RAF (Rapidly Accelerated Fibrosarcoma) signalling pathways revealed the existence of feedforward networks that lead to chemo-resistance for RAF inhibitor-based therapies^82^. Therefore, finding the feedforward incohorencies or negative feedback with buffer action in a given cell signalling network relevant to the context of the treatment can provide early predictions of chemo-resistance and aid in informed patient-specific therapy decisions.

Apart from structure-based prediction, leveraging a network-based approach to systematically discover potential target species has also attracted sustained interdisciplinary interest. For instance, compositional heterogeneity in the TME (Tumor Microenvironment) plays an instrumental role in shaping immunotherapy outcomes^83^. Also, the different clinically observed TME subtypes can be understood as distinct possible *attractors* of the dynamical system comprising the population and concentration dynamics of the different cell types and states underlying the TME^84^. Therefore, from the perspective of dynamical systems theory, the task of identifying the target species that can reprogram an immunotherapy-resistant TME subtype to a favourable one amounts to finding control (override) nodes that steer the state trajectories to a desired attractor.

Establishing global control laws that guarantee convergence to arbitrary attractors for complex networks remains an open problem in control theory. Concepts of minimal driver nodes for ensuring full reachability have been explored before, but placing this in a structural context remained elusive. However, Fiedler et al.^85^, in their seminal work, proposed a structure-theory-based approach that provides a sufficient condition for identifying the set of controller nodes that can drive the network to desired attractors, given that the governing dynamics are dissipative and satisfy a non-zero decay condition. They showed that apart from the source nodes (not reachable from any other nodes), the node set constructed by taking the minimal number of nodes that cover all the existing loops in the network, also known as the feedback vertex sets (FVS) forms the minimal set of controller nodes. Later on, Albert and co-workers^86^ showed that these assumptions are not restrictive and therefore, the FVS-based control strategy can be leveraged with sufficient confidence towards analysing a wide variety of real-world networks ranging from cell signalling, information transmission, to opinion spreading. Recently, Bhattacharya et al.^87^ showed that the cell-state-based networks for solid tumours may also obey the assumptions required for applying FVS-based results and therefore, the FVS-based control strategy can be leveraged towards model-informed target discovery for resistant phenotypes in the context of immunotherapy for solid tumours.

## Beyond Hypermotifs: Embedding, Clustering, and Generalisations

In the previous sections, we introduced and discussed hypermotifs as systematically interconnected motifs that can yield emergent dynamical behaviours. Here, we broaden the view to related mesoscale phenomena that arise when motifs are *embedded* in larger networks, or when recurring patterns appear as *clusters* and *generalisations*. These perspectives complement hypermotifs by showing how (i) a motif’s function can be shaped by its surrounding network context, and (ii) higher-order structure can also emerge through motif aggregation (clustering) or topological expansion (generalization).

### Motif embedding in larger networks

Network motifs often operate while embedded in larger networks. To understand how different types of motif embeddings in a larger complex network affect the motif’s dynamical behaviour, Harlapur et al.^88^ embedded toggle switch and toggle triad in complex larger networks of varying number of nodes (changing network size) and mean connectivity (changing the network density). The results indicate that the correlation coefficient between the steady-state values of two selected nodes in the toggle switch remains largely unchanged when the mean connectivity is fixed, even as the network size (number of nodes) increases. However, if the mean connectivity is increased for a fixed network size, the correlation coefficient between the two nodes decreases. Therefore, the dynamical properties of a toggle switch are affected by network density, rather than the size of the network in which it is embedded. In other words, there is a gradual decrease in the switch-like behaviour of the toggle switch as the network density increases. Similarly, as the in-degree of the nodes increases, the switching behaviour decreases, indicating that the local density around the motif affects the dynamical properties of the motif. However, if there is a huge difference in the in-degree between different nodes in the toggle switch, the switch-like behaviour becomes more prominent and closer to the behaviour of the standalone motif. Further, adding self-inhibitory loops in the motif results in a huge decrease in the switching behaviour as compared to the stand-alone motif behaviour.

Motifs embedded within large complex networks significantly influence the overall dynamic behaviour of the network. For instance, Gross et al.^89^ compare the global stability of complex networks with the local stability of their constituent motifs. It reveals that dense motifs, such as cliques, can exhibit stability properties that differ from those of the entire network. In graph theory, cliques are defined as subsets of nodes where every pair is interconnected. Notably, the study demonstrates that the stable states within dense motifs can enhance the dynamical stability of the entire network and, in some cases, transform locally stable states into globally stable ones.

### Motif generalisations and clustering beyond hypermotifs

In addition to hypermotifs, other higher-level organisational patterns have been described in biological networks, including motif generalisations and motif clustering. Table 2 provides a comparative overview of these concepts in terms of their structures, functional integration, and biological relevance. Notably, hypermotifs involve systematically connected motifs that can give rise to emergent dynamical behaviours beyond those of individual motifs, whereas clustering and generalisations capture different forms of higher-order organisation.

**Table 2:** Comparison of Hypermotifs, Motif Generalisations, and Motif Clustering

Feature	Hypermotifs	Motif Generalisation	Motif Clustering
**Definition**	Structured integration of multiple motifs into a composite, functional unit	Expansion of a single motif by relaxing or extending its topology	Spatial or topological co-occurrence of motifs in a local network region
**Motif Types Involved**	Multiple, possibly different motifs (e.g., FFL + feedback loop)	Single motif type with scalable variation	Same or different motif types
**Structural Focus**	Composition and connectivity between motifs	Internal variation or scaling of one motif	Proximity and density of motifs in the network
**Functional Integration**	Yes — leads to emergent dynamic behaviours	Sometimes — functions are preserved or scaled	Not necessarily — may lack integrated function
**Emergent Properties**	Central to the definition; new dynamics arise from composition	Limited; typically retains known behaviour	Absent or incidental
**Biological Relevance**	Explains higher-order regulation, decision-making, and cell fate control	Models motif families and structural robustness	Reveals modular organisation or regulatory hubs
**Examples**	Feedback loop modulating FFL; toggle switch embedded in a loop	Multi-output FFL; generalised cascade	Cluster of FFLs or feedback loops near a hub transcription factor

**Fig. 9:**
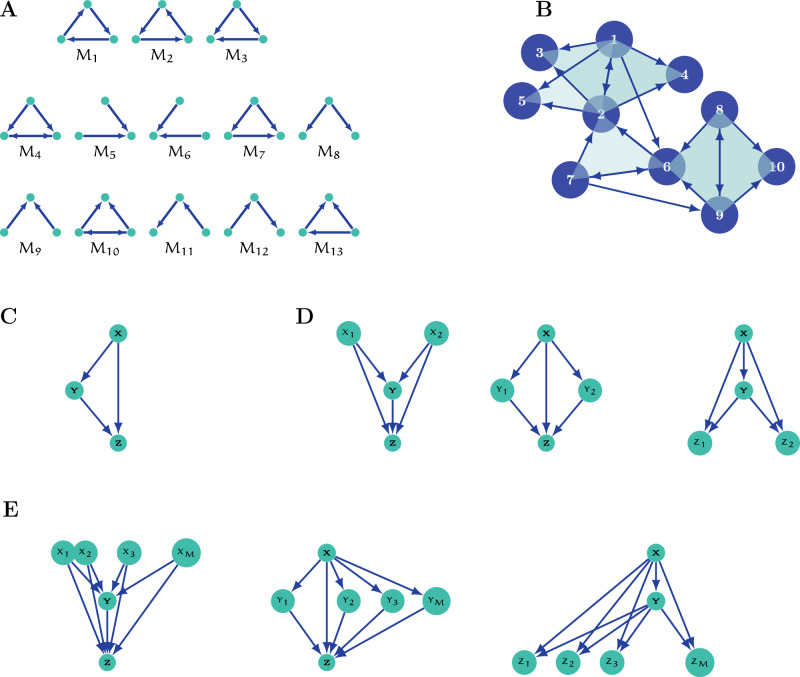
Higher-order network structures and the higher-order network clustering framework. **A** Higher-order structures are captured by network motifs. For example, all 13 connected three-node directed motifs are shown here. **B** Clustering of a network based on motif M7. **C** An FFL motif triad. **D** Three simple generalisations of the three-node FFL motif to the level of four nodes and higher-order motifs in (**E**).

Figure 9 shows how each network motif in Fig. 9A can reveal a distinct higher-order clustering pattern as shown in Fig. 9B^90^. Motif generalisations expand a single motif by relaxing or extending its topology^16,91–93^. Figure 9C shows a three-node FFL, and Fig. 9D shows three four-node generalisations obtained by duplicating one motif *role* (and its connections). Roles capture how nodes repeatedly participate in larger networks; in an FFL, nodes typically serve as a source regulator (${\mathcal{X}}$), an intermediate (${\mathcal{Y}}$), or an output (${\mathcal{Z}}$). Accordingly, duplicating ${\mathcal{X}}$ yields a double-${\mathcal{X}}$ (double-input) FFL, while duplicating ${\mathcal{Y}}$ or ${\mathcal{Z}}$ produces the other two variants (Fig. 9D). This idea can be extended further to produce higher-order motifs such as multi-${\mathcal{X}}$ (multi-input), multi-${\mathcal{Y}}$, and multi-${\mathcal{Z}}$ (multi-output) FFLs as shown in Fig. 9E^16,93^.

A particularly important motif generalisation in transcription is the multi-output FFL. The multi-output FFL has a single input node ${\mathcal{X}}$, a single intermediate node ${\mathcal{Y}}$, and a number of output nodes ${{\mathcal{Z}}}_{1}$, ${{\mathcal{Z}}}_{2}$, …, ${{\mathcal{Z}}}_{M}$, as shown in Fig. 9E. The weights of the network edges correspond to the activation or repression coefficients of each gene. The multi-output FFL extends the classical FFL by enabling precise, tunable temporal ordering of target gene activation via distinct thresholds^94,95^. This allows genes to be expressed in a functional sequence, as seen in E. coli flagellar assembly. It also acts as a persistence detector, filtering out transient signals. Activation and deactivation timing can be independently controlled, making the multi-output FFL a versatile motif for encoding dynamic gene expression programs. Both the prokaryotic (*E. coli*) and eukaryotic (*S. cerevisiae*) sensory transcription networks exhibit the multi-output FFL structure^16,96^.

## Methods

### Discovery methods for hypermotifs and motif-based mesoscale structure

Detecting and characterizing hypermotifs in biological networks requires sophisticated computational methods that can handle the combinatorial complexity of higher-order structures^97–100^. Recent years have seen significant advances in algorithms for hypermotif detection^97–99^. Exact methods for mining higher-order motifs in hypergraphs provide complete enumeration of all instances of specified patterns^97,98^. These approaches extend classical motif-finding algorithms to hypergraph structures, accounting for the increased complexity of many-body interactions. While computationally intensive, exact methods are essential for establishing ground truth and validating sampling approaches. Sampling methods offer scalable alternatives for large networks where exact enumeration is infeasible^98,99^. Recent work has developed efficient sampling algorithms that provide accurate estimates of hypermotif frequencies while maintaining computational tractability. These methods have enabled hypermotif analysis in genome-scale networks and large-scale single-cell datasets. Compression-based inference represents an alternative paradigm for identifying network motifs and hypermotifs^100^. This approach leverages information-theoretic principles to identify recurring patterns that enable efficient network compression. Compression-based methods can discover motifs without requiring pre-specification of pattern types, potentially revealing novel hypermotif structures.

Beyond direct hypergraph mining and compression-based discovery, another major line of work defines hypermotifs as *compositions* of simpler motifs. Accordingly, methods for hypermotif detection have transitioned from simple subgraph enumeration to more advanced frameworks that treat existing motifs as building blocks. The motif assembly framework as proposed in refs. ^3,4^ involves identifying statistically enriched arrangements where multiple network motifs interact or combine to form higher-order structures. The first step in these works is identifying network motifs, upto a specific size *n*, that capture its characteristic structural patterns. A variety of established algorithms are available for detecting such motifs in large-scale networks^1,101–103^. For example, MotifMiner^103^ is a general and extensible toolkit designed for the automatic and efficient detection of motifs across diverse scientific datasets. Built on a core framework for substructure discovery, it supports the analysis of various molecular datasets and can be easily adapted to new domains and customized for specific research needs. FANMOD^101^ is a fast network motif detection tool that uses recently developed algorithms to speed up motif discovery by orders of magnitude compared to earlier methods. The second step is to categorize nodes based on their specific structural roles (e.g., input, bridge, or output) within those motifs and third, quantifying the statistical significance of motif overlaps or linkages. One representative tool for performing such motif-to-motif overlap comparisons is CompariMotif^104^, which compares two lists of protein motifs to identify overlaps and describe the relationships between them. It can compare a motif list with itself, with its reversed version, or with a second motif list. The output is a table listing all pairs of matching motifs, their similarity score (based on information content), and the nature of their relationship. The next step involves computing the Jaccard index for pairs of motif roles to identify nodes that participate in multiple motifs simultaneously. High overlap indicates a frequent combination. To assess the statistical significance of such motif combinations, the observed overlaps are compared against those in randomized “null model” networks using Z-scores and P-values to confirm enrichment. This comparison helps distinguish motif co-occurrence patterns that are functionally meaningful from those that arise due to inherent topological constraints^105,106^. More recent work^107^, casts the motif detection as a sequence-matching problem. By encoding induced subgraphs as symbolic sequences and scoring them with Hidden Markov Models, researchers can identify “noisy" or partial hypermotifs that traditional exact-match algorithms miss.

Apart from hypermotifs, the work in ref. ^90^ focuses on identifying clusters of network motifs. Conceptually, the framework seeks to identify a subset of nodes *S* in a network that is strongly associated with a particular motif *M*. The selection of *S* is guided by two main criteria: (i) the nodes in *S* should collectively participate in a large number of occurrences of motif *M*, and (ii) the motif instances should be well-contained within *S*, minimizing instances where only a subset of the motif’s nodes fall within *S* while the rest lie outside. To formalize this, the framework introduces a higher-order clustering objective defined by the ratio:

$${\Phi }_{M}(S)=\frac{{{\mathrm{cut}}}_{M}(S,\bar{S})}{\min ({{\mathrm{vol}}}_{M}(S),{{\mathrm{vol}}}_{M}(\bar{S}))}$$

Here, $\bar{S}$ denotes the complement of *S*, ${\mathrm{cut}}_{M}(S,\bar{S})$ counts the number of motif instances spanning both *S* and $\bar{S}$, and vol_*M*_(*S*) represents the total number of nodes involved in motif instances entirely within *S*. This formulation generalizes the classic notion of conductance to a motif-aware setting and is also referred to as the motif conductance of S with respect to M.

High-dimensional feedback loops (HiLoop)^41^ is a toolkit for extracting, visualising, and analysing complex interconnected feedback loops within large-scale biological networks. Its key features include: (a) identifying and visualizing feedback structures, (b) assessing the enrichment of specific network configurations, and (c) constructing and simulating dynamic models of selected networks or subnetworks using randomly generated parameter sets. High-feedback loops are detected by finding all cycles of a specific length in the given network using the algorithm of Liu and Wang^108^. Each identified cycle is stored with its net regulatory sign, presence of repression edges, and a list of edges (as node ID pairs). Each feedback cycle is assigned a unique identifier to maintain consistent ordering. To detect high-feedback motifs, the method constructs two auxiliary graphs that represent relationships among cycles: a cycle intersection graph, where each node corresponds to a cycle and edges indicate shared nodes between cycles, and a cycle edge intersection graph, where edges connect cycles sharing at least one common edge. These graphs are used to efficiently identify overlapping cycles that match predefined sign patterns and connectivity rules of specific motifs. If the resulting subnetwork formed by these cycles satisfies the specified size constraints, it is selected as a representative high-feedback module.

In addition to combinatorial and statistical mining, recent work uses graph representation learning to surface candidate motifs and hypermotif-like substructures from prediction tasks. GNN explanation methods (e.g., GNNExplainer^109^, PGExplainer^110^, Gem^111^, and ReFine^112^) typically identify influential edges or nodes, but do not explicitly target motifs or hypermotifs. SubgraphX^113^ is a method to identify network motifs or subgraphs that explain based on GNN predictions. This technique uses Monte Carlo Tree Search^114^ to effectively sample and evaluate different subgraphs derived from an input graph. To quantify the importance of the extracted subgraphs and capture the interactions among different subgraphs, SubgraphX employs Shapley values^115^. The method is used in drug discovery^116^. A drawback of SubgraphX is that the subgraphs identified may not be recurrent or statistically important. MotifExplainer^117^ addresses this by extracting domain-informed motifs first, embedding them through a trained GNN, and then ranking motifs via attention weights to yield motif-based explanations. Complementarily, role discovery aims to identify nodes with similar structural patterns based on feature representations rather than strict graph equivalences. Mapping graph structures into feature spaces derived from node attributes and link patterns enables the comparison of structural similarities. However, approaches like random-walk-based role definitions^118^ remain tied to node identity and fail to capture higher-order connectivity patterns such as network motifs. Another study^119^ introduced Higher-Order Network Embeddings (HONE), a framework for learning node embeddings from network motifs. Instead of relying on manual motif selection, HONE automatically aggregates structural patterns across different motif types and walk depths to capture diverse topological signals. These aggregated features are then compressed into low-dimensional global representations using factorisation or alternative methods such as autoencoders and tensor decompositions. The resulting embeddings are generalizable and can be applied to tasks like role discovery, classification, and link prediction. A summary of computational approaches for hypermotif discovery and motif-based mesoscale organization is provided in Table 3.

**Table 3:** Representative computational methods for hypermotif discovery and motif-based mesoscale structure analysis

Category	Framework	Core idea	Limitations	Refs
Exact discovery	Hypergraph motif mining	Exhaustively enumerate higher-order patterns in hypergraphs to identify all instances of specified hypermotifs.	Poor scalability for large networks.	^97,98^
Sampling-based discovery	Randomized hypermotif sampling	Estimate hypermotif frequencies by probabilistic sampling instead of full enumeration.	Approximation error depends on sampling quality.	^98,99^
Compression-based inference	Information-theoretic discovery	Detect recurrent patterns through their contribution to efficient network compression, without predefined templates.	Can be harder to interpret biologically.	^100^
Motif composition	Coupled-motif / assembly frameworks	Define hypermotifs as enriched combinations of simpler motifs linked by shared nodes or inter-motif edges.	Depends on initial motif detection and overlap rules.	^3,4^
Classical motif detection	FANMOD, MotifMiner	Detect enriched motifs up to a chosen size using subgraph enumeration and statistical testing.	Usually limited to small motif sizes.	^101,103^
Motif overlap analysis	CompariMotif, Jaccard overlap, null-model tests	Quantify shared node participation and test whether motif overlaps are enriched beyond randomized networks.	Sensitive to motif definitions and null-model choice.	^104–106^
Sequence-based detection	HMM-based motif matching	Encode induced subgraphs as symbolic sequences and detect noisy or partial hypermotifs with HMMs.	Requires careful encoding and model specification.	^107^
Mesoscale clustering	Motif conductance	Generalize graph conductance to identify node sets enriched in a given motif while minimizing boundary-spanning motif instances.	Can become costly for large graphs and higher-order motifs.	^90^
Feedback-module extraction	HiLoop	Extract overlapping feedback modules by identifying cycles and their intersections in auxiliary graphs.	Mainly tailored to feedback-rich structures.	^41,108^
Learning-based discovery	SubgraphX, MotifExplainer	Use GNN explanations to identify predictive subgraphs or domain-informed motifs.	Explanations may not correspond to recurrent or enriched motifs.	^109–117^
Higher-order representation learning	HONE	Learn node embeddings from motif-derived higher-order structural features across motif types and walk depths.	Less interpretable than explicit motif discovery.	^119^

### Modeling and Analysis Methods for Hypermotif Circuit Function and Dynamics

Mathematical modeling is central to analyzing hypermotif dynamics and predicting system-level behavior, and a range of complementary frameworks has been used to identify and interrogate hypermotif circuits across biological contexts^3,4,120,121^. ODE models resolve continuous gene-regulatory dynamics, capturing graded responses, temporal transients, and parameter dependence^3^. Boolean models provide a scalable discrete-state alternative that links topology to attractor structure, multistability, and switching among stable states^121,122^. Stochastic analyses further quantify how hypermotif architecture shapes noise propagation and decision reliability, revealing configurations that attenuate fluctuations while preserving responsiveness^120^. In practice, each constituent motif is partitioned into input, internal, and output roles, and hypermotifs are defined by coupling motifs through shared nodes or directed inter-motif edges. Controlled perturbations are then applied at the inputs, and the resulting output behavior is quantified via changes in attractors, bifurcation structure, noise sensitivity, and information-theoretic measures. Random Circuit Perturbation (RACIPE) automates this workflow by constructing ODE models from a given topology and simulating ensembles of randomly sampled kinetic parameters, thereby mapping the dynamical repertoire implied by network structure^123^. Complementarily, Dynamic Signatures Generated by Regulatory Networks (DSGRN) provides a parameter-free, combinatorial abstraction that partitions parameter space into regions with distinct qualitative dynamics (e.g., multistability and oscillations) and assigns each region a dynamic “signature,” enabling global characterization and design ranking for robustness^124^. At the network level, where interaction structure may change across conditions or time, DyNet^125^ offers a Cytoscape-based workflow for visualizing and synchronizing multi-state molecular interaction networks and for identifying the most “rewired” nodes across states.

These modeling and screening tools have motivated a growing body of computational work that searches for small regulatory circuits embedded within large gene networks and links them to specific dynamical phenotypes. For example, recent studies have focused on identifying circuits that generate multistability, synchronization or oscillations^42,126–128^. A previous study^42^ identified three-node circuits capable of stepwise transitions between four states, shedding light on regulatory mechanisms in T-lymphocyte development. Schaerli et al.^127^ studied stripe-forming circuits and found that incoherent FFLs and a simple two-node motif with both activation and inhibition were central to spatial patterning. However, these approaches can be limited by restricted parameter-space exploration, the lack of quantitative metrics for ranking or comparing circuit functions, and difficulty in isolating how coupling between motifs reshapes dynamics beyond what isolated motifs predict. Addressing these gaps, Clauss et al.^129^ propose a framework to identify gene-circuit motifs and their couplings (hypermotifs) using a quantitative functional metric. By systematically simulating all non-redundant four-node transcriptional networks (approximately 60,000 circuits) over broad parameter ensembles, they quantify both the contributions of individual two-node motifs and the impact of coupling within larger structures. Steady-state expression distributions are extracted from the ODE ensembles, and enriched motifs and motif couplings are linked to specific expression patterns, with networks ranked via a score based on the Euclidean distances between cluster centroids. In a complementary direction focused on system-level functionality, Zhu et al. develop a methodological pipeline for analyzing resilience in flow-weighted networks by converting cascading-failure dynamics into a hypergraph representation, in which hyperedges encode directed failure dependencies between nodes, and then applying percolation theory to characterize phase transitions during network degradation^36^. In their construction, the hypergraph matrix *H* is obtained by recording whether a perturbation at node *i* induces failure at node *j*, yielding a dynamics-informed substrate for higher-order pattern discovery. After the percolation transition, they define *stable hyper-motifs* as persistent, localized interdependency patterns among cascading failures within the surviving components, and introduce a normalization of hyper-motif counts to enable comparison across networks. This framework provides a functional, mechanism-linked route for identifying a small set of critical hyper-motifs that can disproportionately shape macroscopic resilience outcomes.

#### From single cell transcriptomics to digital twins

**Fig. 10:**
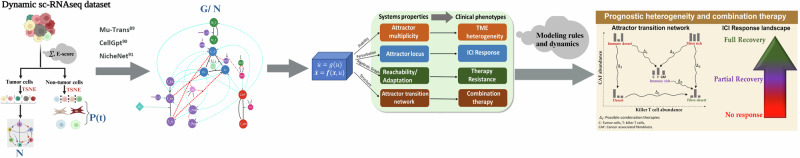
Topology informed digital twin framework for patient specific immunotherapy decision support. A schematic demonstrating the conceptual framework for construction of a digital twin using a topology-informed systems formalism and patient-specific dynamic single-cell transcriptomic data in the context of immunotherapy for solid tumors. Based on the dataset and pre-decided granularity, the network is constructed through integrating different statistical toolboxes for identifying a diverse set of cellular interactions (Transition, paracrine, autocrine, killing, regulatory inhibition, activation, and secretion)^131 -- 133^. The cardinality of each cluster represents the density of each cell states---this can be leveraged for fitting the underlying mechanistic model parameters (for instance, proliferation, death, interaction rates). Further, we evaluate the closed-form conditions in^87^ determining relevant therapy decisions, in the given model and parametric context to aid in patient-specific therapy decisions.

One of the most exciting applications of a topology-informed treatment of complex systems in biology lies in its potential for designing systematic pipelines for personalised treatment. It is in this context that the traditional mathematical modelling, dynamical systems theory, and the abundance of open-source, publicly available single-cell transcriptomics data can converge towards designing personalised digital twins that can provide a go/no-go decision for an individual patient in the context of a particular treatment regimen. Leveraging the existing disease-specific single-cell dataset, it is possible to construct the cell state transition networks^130,131^. Further, additional toolsets such as CellChat and Niche-Net can also be applied to identify potential paracrine interactions between cellular states^132,133^. The secretome analysis in the single cell resolution can potentially provide information about the cell state-cell interactions via secreted agents such as cytokines, and chemokines. Taken together, this aids in constructing the disease-specific complex cell-state network. Subsequently, exploiting the wealth of dynamical systems theory and structure theory, the disease-specific clinical features of interest can be mapped to select properties of the underlying dynamical system (phenotype-systems mapping) constructed from the cell state network. Further, the well-behaved, generalizable properties, such as concave proliferation and bounded interactions across a wide range of cellular and subcellular kinetics (such as logistic/Gompertz/Simeone proliferation laws or Michaelis-Menten/Hill kinetics), render a *kinetic-agnostic* treatment of biological networks possible to a great extent. For instance, it may be possible to predict just from the network structure whether a particular patient (with a particular network structure) would respond to immunotherapy/chemotherapy. If negative, then there may be ways to systematically identify an appropriate target to circumvent the resistance^81,87^. Therefore, a patient-specific transcriptomic signature can inform the patient-specific networks (cell state, gene regulatory networks depending on the disease context) with the cell state densities which can further be fitted against the underlying Quantitative Systems Pharmacology model (QSP). Further, the generic closed-form conditions governing the properties of clinical interests (sensitivity/ resistance to therapy, existance and systematic identification of suitable combination therapy) may be evaluated in the given context of the QSP model and fitted parameters to provide the patient-specific treatment decisions and dose levels (Fig. 10). A summary of modeling and analysis frameworks for hypermotif circuit function and dynamics is presented in Table 4.

**Table 4:** Representative modeling and analysis methods for hypermotif circuit function and dynamics

Category	Framework	Core idea	Limitations	Refs
Continuous dynamical modeling	ODE models	Use ordinary differential equations to study graded responses, temporal transients, bifurcations, and parameter dependence.	Requires kinetic assumptions and many uncertain parameters.	^3^
Discrete dynamical modeling	Boolean models	Use logical update rules to connect hypermotif topology with attractors, switching, and multistability.	Ignores graded kinetics and fine timing effects.	^121,122^
Noise-aware analysis	Stochastic models	Quantify how hypermotif organization shapes fluctuation propagation, noise buffering, and decision reliability.	Hard to analyze and scale for larger circuits.	^120^
Parameter-ensemble analysis	RACIPE	Sample large parameter ensembles for a fixed topology to map the range of admissible dynamical behaviors.	Computationally intensive and less mechanistically specific.	^123^
Parameter-space abstraction	DSGRN	Partition parameter space into regions with distinct qualitative dynamics such as oscillation or multistability.	Coarse-grained rather than trajectory-level.	^124^
State-dependent network analysis	DyNet	Compare networks across conditions or time points to identify rewired nodes and changing interaction patterns.	Focuses on rewiring more than explicit hypermotif function.	^125^
Functional circuit screening	Large-scale simulation and ranking	Simulate many candidate circuits and rank motifs or couplings by quantitative functional scores.	Sensitive to scoring metric and computational budget.	^42,126–129^
Dynamics-informed hypergraph analysis	Hypergraph/percolation-based resilience analysis	Build hypergraphs from cascading-failure dependencies and identify stable hypermotifs linked to resilience transitions.	Specialized to failure-propagation settings.	^36^

## Future Perspectives

This review highlights frameworks that analyse how network motifs combine into hypermotifs and the potential emergent behaviours they exhibit across biological networks. Such approaches reveal mesoscale organisational patterns, functional modules, and underlying design principles that govern complex regulatory systems. In transcriptional networks, interconnected positive feedback loops and hub-like hypermotifs constrain the number of accessible attractor states, whereas serial and cyclic networks with lower node centrality increase the diversity of stable states, promoting non-genetic phenotypic heterogeneity. Layered coherent type 1 FFLs (C1FFLs) in TGF-*β*-induced EMT demonstrate how hypermotif architectures integrate upstream signals, filter noise, and produce intermediate or hybrid states, ensuring robust cell fate transitions in cancer. In developmental gene regulatory networks, motif identity and gene positions within motifs can predict expression patterns and highlight key regulators of critical developmental transitions. Positive feedback circuits are highly enriched, suggesting evolutionary selection for circuits that provide delay in response while buffering noise, whereas cascades of FFLs are less common, likely due to trade-offs between response time and signal fidelity.

Future work should experimentally validate these motifs and hypermotif predictions across diverse biological contexts, confirming their roles in shaping cellular behaviours and intermediate states. Extending analyses to larger, more complex regulatory networks and incorporating stochastic, noise-driven dynamics could reveal how multistability, robustness, and emergent properties are maintained in physiologically relevant systems. Generalizing hypermotifs to hypergraphs would allow exploration of multi-node interactions, such as protein complexes or collaborative regulatory modules, while comparing the functional contributions of simple larger motifs with hypermotifs could uncover new organisational principles. Additionally, investigating how hypermotif architectures regulate morphogen signalling, temporal activation of transcription factors, and other dynamic cellular processes could provide further insights into the design principles of development and disease. These systems-level insights may guide the design of synthetic multistable circuits and inform strategies to control cell-fate decisions and other complex biological behaviours.

## Data Availability

No datasets were generated or analysed during the current study.
